# Potential association of plasma lysophosphatidic acid (LPA) species with cognitive impairment in abstinent alcohol use disorders outpatients

**DOI:** 10.1038/s41598-020-74155-0

**Published:** 2020-10-13

**Authors:** Nuria García-Marchena, Nieves Pizarro, Francisco J. Pavón, Miriam Martínez-Huélamo, María Flores-López, Nerea Requena-Ocaña, Pedro Araos, Daniel Silva-Peña, Juan Suárez, Luis J. Santín, Rafael de la Torre, Fernando Rodríguez de Fonseca, Antonia Serrano

**Affiliations:** 1 Laboratorio de Medicina Regenerativa, Unidad de Gestión Clínica de Salud Mental, Instituto de Investigación Biomédica de Málaga (IBIMA), Hospital Regional Universitario de Málaga, Avda. Carlos Haya 82, sótano, 29010 Málaga, Spain; 2 Institut D, Investigació en Ciències de la Salut Germans Trias i Pujol (IGTP), Unidad de Adicciones-Servicio de Medicina Interna, Campus Can Ruti, Carrer del Canyet s/n, 08916 Badalona, Spain; 3 Integrative Pharmacology and Systems Neurosciences Research Group, Programa de Investigación en Neurociencias, Institut Hospital del Mar d’Investigacions Mediques (IMIM), Dr. Aiguader 88, 08003 Barcelona, Spain; 4 Unidad de Gestión Clínica del Corazón, Instituto de Investigación Biomédica de Málaga (IBIMA), Hospital Universitario Virgen de la Victoria de Málaga, Malaga, Spain; 5 Centro de Investigación Biomédica en Red de Enfermedades Cardiovasculares (CIBERCV), Instituto de Salud Carlos III, Madrid, Spain; 6 Departamento de Nutrición, Ciencias de los Alimentos y Gastronomía, Facultad de Farmacia y Ciencias de los Alimentos, Universidad de Barcelona, Barcelona, Spain; 7 Departamento de Psicobiología y Metodología de las Ciencias del Comportamiento, Instituto de Investigación Biomédica de Málaga (IBIMA), Facultad de Psicología, Universidad de Málaga (UMA), Malaga, Spain

**Keywords:** Molecular biology, Neuroscience

## Abstract

Lysophosphatidic acid (LPA) species are bioactive lipids participating in neurodevelopmental processes. The aim was to investigate whether the relevant species of LPA were associated with clinical features of alcohol addiction. A total of 55 abstinent alcohol use disorder (AUD) patients were compared with 34 age/sex/body mass index-matched controls. Concentrations of total LPA and 16:0-LPA, 18:0-LPA, 18:1-LPA, 18:2-LPA and 20:4-LPA species were quantified and correlated with neuroplasticity-associated growth factors including brain derived neurotrophic factor (BDNF), insulin-like growth factor-1 (IGF-1) and IGF-2, and neurotrophin-3 (NT-3). AUD patients showed dysexecutive syndrome (22.4%) and memory impairment (32.6%). Total LPA, 16:0-LPA, 18:0-LPA and 18:1-LPA concentrations, were decreased in the AUD group compared to control group. Total LPA, 16:0-LPA, 18:2-LPA and 20:4-LPA concentrations were decreased in men compared to women. Frontal lobe functions correlated with plasma LPA species. Alcohol-cognitive impairments could be related with the deregulation of the LPA species, especially in 16:0-LPA, 18:1-LPA and 20:4-LPA. Concentrations of BDNF correlated with total LPA, 18:2-LPA and 20:4-LPA species. The relation between LPA species and BDNF is interesting in plasticity and neurogenesis functions, their involvement in AUD might serve as a biomarker of cognitive impairment.

## Introduction

Alcohol use disorders (AUD) are one of the main global health problems, with a worldwide impact on individuals and society. They cause a significant medical burden in prevention and treatment effort^1,2^. Among the medical consequences related to alcohol dependence we can highlight the prevalence of nutritional deficiencies, hepatic and liver damage^3,4^ and the appearance of comorbid psychopathological lifetime complications^5^. Moreover, cognitive impairment associated to AUD is considered one of the main factors for the development of any type of dementia^6^. The presence of comorbid medical conditions during AUD could contribute to the lack of control over drinking aggravating the medical treatment^7^.

While major depressive disorders and anxiety are the most prevalent psychopathological comorbid disorders in alcohol use population^8–10^, cocaine and cannabis abuse are the most common lifetime comorbid substance disorders associated to AUD^11^. Moreover, chronic alcohol consumption could modulate neurogenesis and produce distortions on the central nervous system causing neurocognitive impairment^12,13^. Thus, functional circuits involved on these medical consequences of alcohol include those related to cognition (such as prefrontal cortex-involving circuits) and emotional processing (such as limbic-amigdalar circuits). The most common cognitive alterations in alcohol dependence patient are those related with executive functions, episodic and visuoconstructive memory and emotion^4,14^. In AUD patients, the measurable neuropsychological damage belonging to executive functions have been found impaired in those tasks related to inhibition, flexibility, deduction of rules, organization and planning^15,16^. Recently, we have linked AUD associated with mild and severe cognitive impairment and to a dysfunctional signaling of both, the insulin growth factor (IGF) and the brain derived neurotrophic factor (BDNF), a member of the neurotrophin family of growth factors^17–19^. These findings suggest that at least partially, monitoring BDNF signaling might help to identify early stages of cognitive impairment in AUD patients. However, in these studies neither IGF-1 nor BDNF were found to be associated to any additional comorbidity, especially psychiatric entities such as depressive and psychotic disorders that it is known to contribute to cognitive impairment^17^.

In an attempt to further explore biomolecules that might mediate the effects of alcohol on psychiatric comorbidity and cognitive impairment, we focused our research on lysophosphatidic acid (LPA), an ubiquitous signaling lipid involved in neurodevelopment^20,21^ and emotional behavior, including anxiety^21,22^. LPA has the simplest structure of all glycerophospholipids in serum and is highly expressed in the brain^23^. The term LPA refers to a group of different chemical species where the glycerophospholipid moiety is associated to several different carbon acyl chains (i. e. 16-, 18- and 20-LPA), being the species mono-unsaturated oleic acid (18:1-LPA), and poly-unsaturated linoleic acid (18:2-LPA) and arachidonic acid (20:4-LPA) the more abundant in human serum^24,25^. LPA participates in brain cortex formation, cell proliferation, differentiation and survival functions through the activation of distinct G-protein-coupled receptors^20,26,27^, although there are evidences that LPA participates in apoptosis processes through oxidative stress^28^. In addition, LPA also is involved in adult neurogenesis^29,30^. These important roles in neurodevelopment and plasticity have suggested that abnormalities in LPA signaling might be related to neurodevelopmental disturbances and neuropsychiatric diseases as schizophrenia, Alzheimer’s diseases or autism^31,32^. Recent preclinical studies have also linked LPA to depression, since genetically modified animals lacking LPA1 receptors exhibits an anxious-depressive phenotype^33^ associated with high alcohol intake^34^. Because of the previously described involvement of LPA in both alcohol intake and depression, and the participation of these lipids in neural plasticity events, it is feasible to suspect a potential role for LPA species in comorbidities associated with AUD.

Since preclinical studies have suggested a potential role of LPA and its LPA1 receptor as vulnerability factors for excessive alcohol intake, depression and cognitive impairment, we further explore whether this association might also be present in AUD patients. Thus, the main aim of this descriptive clinical study was to characterize the plasma concentrations of LPA species in a chronic alcohol dependence context and to explore if they correlate with the associated psychiatric (i.e. affective disorders) and neuropsychological (cognitive impairment) comorbidities. Additionally, because previous studies have reported the existence of sexual dimorphism in the biological activity of LPA with a greater presence in healthy women compared with men^30,35^, the present study was performed in male and female patients to examine the plasma concentration of LPA species according to sex.

## Materials and methods

### Participants and recruitment

The present study included 89 Caucasian volunteers divided into two groups: 55 abstinent alcohol use disorders patients (AUD group) in outpatient treatments and 34 control subjects (Control group) matched by age, body mass index (BMI) and proportion of sex with the alcohol group. Patients were recruited at the *Hospital Universitario 12 de Octubre* (Madrid, Spain) and *Centro provincial de Drogodependencias* (Malaga, Spain). Control participants were included from databases of healthy subjects willing to participate in medical research projects from *Hospital Universitario 12 de Octubre* (Madrid, Spain) and *Hospital Regional Universitario de Malaga* (Málaga, Spain).

To be eligible for the present study, participants had to meet the following inclusion criteria: ≥ 18–65 years of age, and abstinence from alcohol for at least 4 weeks. The exclusion criteria included: personal history of long-term inflammatory diseases or cancer, cognitive or language limitations, pregnant or breast-feeding women, and infectious diseases. With regard the control group, participants with psychiatric disorders in Axis I were also excluded.

### Clinical and neuropsychological assessments

Substance use disorders and other psychiatric disorders were diagnosed according to the DSM-IV-TR criteria^36^ using the Spanish version of the Psychiatric Research Interview for Substance and Mental Disorders (PRISM)^36,37^. PRISM is a semi-structured interview with good psychometric properties in the evaluation of substance use disorders and in the main psychiatric comorbid disorders related to substance use population^37,38^.

The cognitive assessment was performed with two scales, the Spanish Versions of the Frontal Assessment Battery (FAB)^39^ and the Memory Failures Everyday (MFE)^40^. The FAB test is useful for a screening of a frontal lobe dysfunction evaluation. The total score was obtained from 0 to 18 evaluating the respectively subdomains: prehension, go-no-go, conflicting, Luria motor and lexical fluency; a cut-off point less than 16 separate normal from mild dysexecutive deficits, and a cut-off point less than 13 separate mild and severe dysexecutive syndrome^39^. The MFE questionnaire is formed by 30 items and is useful to evaluate the lack of memory in daily life. The cognitive complaints scores over 36 are related with memory deficits^40^.

### Collection of plasma samples

Plasma sample collection was based on previous studies about lipid mediators in addiction and comorbid disorders^41^. Blood samples were obtained in the morning after fasting for 8–12 h (prior to the psychiatric interviews) by experienced nurses. Venous blood was extracted into 10 mL K_2_ EDTA tubes (BD, Franklin Lakes, NJ, USA) and immediately centrifuged at 2200×*g* for 15 min (4 °C) to obtain plasma. The plasma samples were individually assayed to detect infectious diseases using commercial rapid tests for HIV, hepatitis B, and hepatitis C (Strasbourg, Cedex, France). Plasma samples were individually stored at – 80 °C until further analyses.

### Analysis of LPA species

The LPA species of saturated fatty acids palmitic acid (16:0-LPA) and stearic acid (18:0-LPA), the LPA of the monounsaturated fatty acid oleic acid (18:1-LPA), and the LPA species of the polyunsaturated fatty acids linoleic acid (18:2-LPA) and arachidonic acid (20:4-LPA) were determined using an extraction protocol followed by LC–MS–MS separation and quantification. Briefly, 0.2 mL of plasma were spiked with 100 ng of a mathanolic solution of 17:0-LPA (IS). A liquid–liquid extraction was performed after the addition of 200 μL of butanol. The organic phase was evaporated and reconstituted in 100 μL of mobile phase (80A:20B, see below) prior to analysis.

Stock solutions (100 μg/mL) for each analyte were independently prepared by diluting adequate amounts of standards in methanol. The working solutions were prepared by mixture of the stock solutions and dilution in methanol. The linearity of calibration curves containing the following concentrations for all the target analytes: 0.2, 0.5, 1, 1.5, 2, 4, 6, 8, 10 μg/mL was verified being the coefficient of determination r^2^ > 0.99 in all cases.

Before the quantification of real samples and in order to verify matrix effect and recovery of the analytical method for each analyte, calibration curves were prepared in both plasma and water samples. In all cases, matrix effects lower than 6% and recoveries higher than 66% were achieved. At this point, calibration curves to perform quantification of real samples were prepared in water, and were added in duplicate in each analytical batch.

The procedure of lipid analysis in plasma was performed by a validated method previously described in clinical samples^41^. Quantification of LPA species in human plasma was performed using an ACQUITY UPLC system (Waters Associates, Milford, MA, USA) for the chromatographic separation coupled to a triple quadrupole (Xevo TQ-S micro) mass spectrometer provided with an orthogonal Z-spray-electrospray interface (ESI) (Waters Associates, Milford, MA, USA). The drying and nebulizing gas was nitrogen. The desolvation gas flow was set to 1200 L/h and the cone gas flow to 50 L/h. A capillary voltage of 3 kV was used in negative ionization mode. The nitrogen desolvation temperature was set to 600 °C and the source temperature to 150 °C. Collision gas was argon and the injection volume was 5 μL.

The chromatographic separation was achieved at 30 °C using an ACQUITY UPLC BEH C18 column (2.1 × 100 mm × 1.7 µm) (Waters Associates, Milford, MA, USA), at a flow rate of 300 µL/min. Mobile phase A was ammonium formate 1 mM with formic acid (0.01% v/v) dissolved in methanol. Mobile phase B was ammonium formate 1 mM with formic acid (0.01% v/v) in water. A gradient program was employed for the separation of the analytes; the percentage of mobile phase B linearly changed as follows: 0 min, 20%; 0.2 min, 20%; 6 min, 10%; 6.5 min, 10%; 7 min, 20%; 8 min, 20%. Total run time was 8 min. Analytes were determined by a Selected Reaction Monitoring (SRM) method by acquiring two transitions for each compound as specified (Supplementary Table S1). The most specific transition was selected for quantitative purposes. MassLynx software V4.1 and TargetLynx XS were used for data management. Finally, the LPA species plasma concentrations were recalculated to molar concentration (nmol/L).

### Analysis of neurotrophic factors

Plasma concentrations of BDNF, IGF-2 and NT-3 were determined using different enzyme-linked immunosorbent assay (ELISA) according to the manufacturer’s instructions: human BDNF SimpleStep ELISA Kit (#ab212166, Abcam, Cambridge, UK) human IGF-2 Quantikine ELISA Kit (#DG200, R&D Systems, Minneapolis, MN, USA) and NT-3 ELISA Kit (#EHNTF3, ThermoFisher Scientific, Alcobendas, Madrid, Spain). To perform the ELISA protocols we used 50 μL of plasma as described previously^17^ and plasma concentrations of IGF-1 were estimated by double antibody radioimmunoassay. Plasma fractions were incubated with 125I-IGF-1 at 4 °C for 24 h with IGF-1 antiserum (UB2-495). In each assay a calibration curve and internal controls were included. The BDNF, IGF-1, IGF-2 and NT-3 plasma concentration were recalculated to molar concentration (nmol/L).

### Statistical analysis

All data in the tables are expressed as number and percentage of subjects [N (%)] or mean and standard deviation (SD). The significance of differences in categorical and normal continuous variables was determined using Fisher’s exact test (chi-square test) and Student’s *t* test, respectively.

Multiple analysis of covariance (ANCOVA) was performed to indicate the relative effects of explanatory variable (i.e., lifetime alcohol use disorders, cognitive impairment) on the plasma concentrations of molecular LPA species, controlling for additional independent variables and covariates (e.g., sex, age and BMI). Because we used factors with two levels and there were not significant interactions between factors in the ANCOVA models, post hoc tests for multiple comparisons were not performed. Correlation analyses were performed using the Spearman’s coefficient (rho) (Plasma concentrations of LPA species and MFE or FAB scores) and correlation analyses using the Pearson’s coefficient (r) in logarithm (10)-transformation concentrations of LPA species and growth factors (BDNF, IGF-1 and IGF-2, and NT-3) to ensure statistical assumption for positive skewed distribution. The statistical analyses were carried out with the GraphPad Prism version 5.04 (GraphPad Software, San Diego, CA, USA), and IBM SPSS Statistical version 22 (IBM, Armonk, NY, USA). A *p* value < 0.05 was considered statistically significant.

### Ethics statements

Written informed consents were obtained from each participant after a complete description of the study. All the participants had the opportunity to discuss any questions or issues. The study and protocols for recruitment were approved by the Ethics Committee of the Hospital Regional Universitario de Malaga (CP14/00173, CP14/00212 and PI13/02261) in accordance with the Ethical Principles for Medical Research Involving Human Subjects adopted in the Declaration of Helsinki by the World Medical Association (64th WMA General Assembly, Fortaleza, Brazil, October 2013) and Recommendation No. R (97) 5 of the Committee of Ministers to Member States on the Protection of Medical Data (1997), and Spanish data protection act [Regulation (EU) 2016/679 of the European Parliament and of the Council 27 April 2016 on the protection of natural persons with regard to the processing of personal data and on the free movement of such data, and repealing Directive 95/46/EC (General Data Protection Regulation). All collected data were given code numbers in order to maintain privacy and confidentiality.

## Results

Table 1 shows a socio-demographic description of the 89 participants of both gender included in this study. We selected 55 patients in abstinence from AUD outpatient programs and 34 healthy control subjects matched for sex, age and BMI with AUD patients. Significant differences were observed between the two sample groups with respect to marital status (p < 0.05) education degree (p < 0.05) and occupation (p < 0.001). The mean age of the AUD group was 48 years and the 82% of the participants were men with a BMI of 26. AUD group displayed higher prevalence of separations and divorces, secondary educational level, and unemployment rate than the control group.

**Table 1:** Socio-demographic characteristics of the sample.

Variables	Total sample N = 89		*p *value
	Control group N = 34	AUD group N = 55	
**Age (mean ± SD)**			
Years	46.9 ± 8.9	47.7 ± 7.7	0.643^a^
**Body mass index (mean ± SD)**			
Kg/m^2^	26.8 ± 4.2	26.1 ± 3.8	0.433^a^
**Sex [N (%)]**			
Women	9 (26.5)	10 (18.2)	0.428^b^
Men	25 (73.5)	45 (81.8)	
**Marital status **[*N* (%)]			
Single	9 (26.5)	13 (23.6)	**0.040**^b^
Cohabiting	19 (55.9)	17 (30.9)	
Separated	6 (17.6)	23 (41.8)	
Widow	–	2 (3.6)	
**Education degree [N (%)]**			
Elementary	2 (5.9)	17 (30.9)	**0.014**^b^
Secondary	18 (28.1)	25 (45.5)	
University	14 (41.2)	13 (23.6)	
**Occupation [N (%)]**			
Employed	34 (100)	42 (41.2)	** < 0.001**^b^
Unemployed	–	60 (58.8)	

*AUD* alcohol use disorders.

^a^*p* value was calculated with Student’s t test.

^b^*p* value was calculated with Fischer’s exact test or chi-squared test.

Bold values are statistically significant for *p* < 0.05.

### Alcohol-related variables and cognitive deficits in AUD group

The variables related to AUD group were evaluated and described in Table 2. The mean age at first drink of alcohol was 15.3 years, while the average age of the AUD onset was 30.2 years with 14.8 years of problematic alcohol use. The mean of addiction criteria was 8 (based on DSM-5) and they had a length of 79 days of abstinence at the moment of the evaluation.

**Table 2:** Clinical characteristics of the AUD group.

Variables	AUD group N = 55
**Age at first alcohol use [mean (SD)]**	
Years	15.3 (3.1)
**Age at onset of AUD [mean (SD)]**	
Years	30.2 (10.5)
**Length of AUD diagnosis [mean (SD)]**	
Years	14.8 (10.0)
AUD severity criteria [mean (range)]	8.3 [1–11]
**Length of abstinence [mean (range)]**	
Days	79 [30–300]
Smoking [*N* (%)]	42 (74.2)
**Comorbid substance use disorders [N (%)]**	
Cocaine	20 (22.5)
Cannabis	4 (4.5)
Sedatives	2 (2.2)
**Comorbid psychiatric disorders N (%)]**	
Mood	24 (27.0)
Anxiety	19 (21.3)
Borderline	5 (5.6)
ADHD	1 (1.1)
Psychotic	8 (7.8)
Antisocial	5 (4.9)
**Psychiatric medication [N (%)]**	
No	8 (14.5)
Antidepressants	29 (52.7)
Anxiolytics	23 (41.8)
Anticraving	16 (29.1)
Disulfiram [*N* (%)]	39 (70.9)
**Frontal cognition deficit (FAB) [N (%)]**	
No	35 (63.6)
Mild cognitive impairment	14 (15.7)
Severe impairment deficit	6 (6.7)
**Memory deficit (MFE) [N (%)]**	
No	26 (29.2)
Mild memory deficit	24 (27.0)
Severe memory deficit	5 (5.6)

*AUD* alcohol use disorders, *ADHD* attention deficit hyperactivity disorder.

Regarding other abused substances, a 74.2% of the AUD group were smokers and there was a high prevalence of other substance use disorders (SUDs) (41.8%), being cocaine the most prevalent substance use (22.5%). In addition to the SUDs, there was observed an elevated prevalence of other psychiatric disorders (63.6%). Thus, lifetime mood and anxiety disorders were diagnosed in a 27% and 21.3% of AUD group, respectively. Unlike the control participants, the 85.5% of the abstinent alcohol patients received psychiatric medication during the last 12 months: antidepressants (52.7%), anxiolytics (41.8%) and anticraving (29.1%). Finally, the 70.9% of the AUD group were treated with *disulfiram*.

The neuropsychological evaluation revealed that a 22% of the AUD group showed some deficits related to frontal cognition (assessed with FAB); 33% of them suffer memory deficits (assessed with MFE) and 31% showed some impairment of both frontal cognition and memory deficits.

### Plasma concentrations of LPA species in abstinent alcohol patients

The impact of the alcohol dependence was studied in the total sample using a two-way ANCOVA with “group” (AUD group and control group) and “sex” as factors, and age and BMI as covariates (Supplementary Table S2).

Plasma concentration of the total LPA was significantly affected by “group” and by “sex” factors, but there was no interaction effect between both factors. Plasma concentration of total LPA was significantly affected by the factor “group” [*F*_(1,82)_ = 4.629; *p* = 0.034]. There was a significant reduction in total LPA plasma concentration in the AUD group compared with the control group [60.847 (95% CI 52.190–69.503) nmol/L and 74.922 (95% CI 65.190–84.512) nmol/L, respectively]. Regarding LPA species, plasma concentration of 16:0-LPA [*F*_(1,82)_ = 5.640; *p* = 0.020], 18:0-LPA [*F*_(1,82)_ = 5.166; *p* = 0.026] and 18:1-LPA [*F*_(1,82)_ = 7.114; *p* = 0.009] were significantly affected by the factor “group”. Specifically, 16:0-LPA, 18:0-LPA and 18:1-LPA plasma concentrations were significantly lower in the AUD group than in the control group [16:0-LPA: 8.094 (95% CI 6.979–9.210) nmol/L and 10.096 (95% CI 8.861–11.332) nmol/L; 18:0-LPA: 2.958 (95% CI 2.727–3.199) nmol/L and 3.372 (95% CI 3.105–3.639) nmol/L; and 18:1-LPA: 5.998 (95% CI 25.258–6.738) nmol/L and 7.489 (95% CI 6.670–8.308) nmol/L; respectively]. By contrast, 18:2-LPA and 20:4-LPA plasma concentrations were not affected by the “group” factor or interaction effect between “group” and “sex” factors.

**Figure 1:**
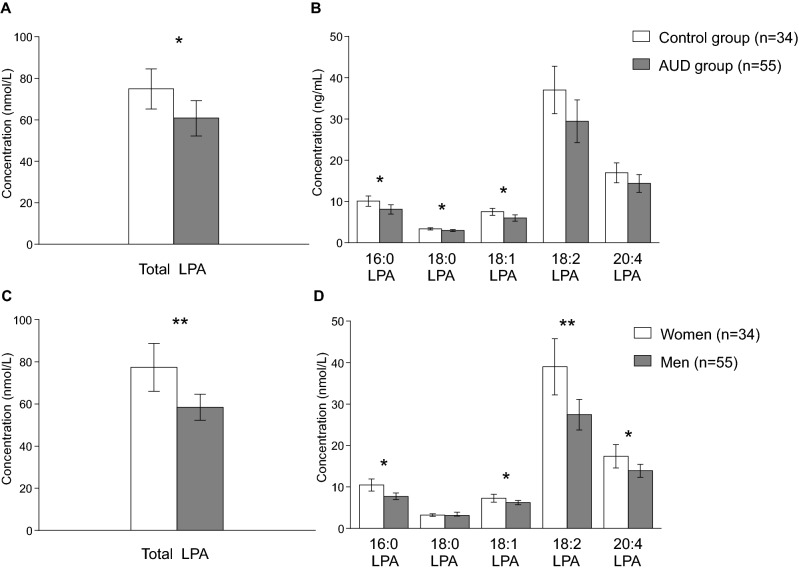
Plasma concentrations of LPA species in the sample according to the group and sex. (**A**) Bars are estimated marginal means and 95% confidence intervals (95%) representing total LPA (nmol/L) according to the group; (**B**) bars are estimated marginal means and 95% confidence intervals (95%) representing LPA species (nmol/L) according to the group; (**C**) bars are estimated marginal means and 95% confidence intervals (95%) representing total LPA (nmol/L) according to the sex; (**D**) bars are estimated marginal means and 95% confidence intervals (95%) representing LPA species (nmol/L) according to the group. Data were analyzed by two-way analysis of covariance (ANCOVA) and *p < 0.05 and **p < 0.010 denote a significant main effect of group factor or sex.

Regarding the impact of “sex”, the plasma concentration of total LPA [*F*_(1,82)_ = 8.377; *p* = 0.005], 16:0-LPA [*F*_(1,82)_ = 10.539; *p* = 0.002], 18:2-LPA [*F*_(1,82)_ = 8.755; *p* = 0.004] and 20:4-LPA [*F*_(1,82)_ = 4.548; *p* = 0.036] were significantly different between men and women. Thus, women had higher total LPA concentrations than men [77.360 (66.023–88.698) nmol/L and 58.409 (95% = 52.205–64.613) nmol/L, respectively]. Similarly, we observed significantly higher concentrations of 16:0-LPA, 18:2-LPA and 20:4-LPA in women than in men [16:0-LPA, 10.465 (9.004–11.926) nmol/L and 7.726 (95% CI 6.926–8.525) nmol/L; 18:2-LPA, 39.012 (95% CI 32.234–45.790) nmol/L and 27.429 (95%CI 23.720–31.138) nmol/L; and 20:4-LPA, 17.405 (95% CI 14.572–20.237) nmol/L and 13.916 (95% CI 12.916–15.466) nmol/L; respectively] (see Supplementary Table S3). Estimated marginal means for “group” and “sex” factors are represented in Fig. 1.

### Plasma concentrations of LPA species in comorbid psychiatric disorders in abstinent alcohol patients

As shown in the clinical description of the sample (Table 2), mood and anxiety disorders were the most prevalent comorbid psychiatric disorders in the AUD group. Thus, we examined the effect of mood and anxiety comorbid disorders in total LPA and LPA species in the AUD group using a two-way ANCOVA with “comorbidity/mood or anxiety disorders” (comorbid subgroup and non-comorbid subgroup) and “sex” as factors, and age and BMI as covariates. However, we did not observe main effects or interaction effects on LPA concentrations (Supplementary Table S4, S5).

### Plasma concentrations of LPA species in cognitive impairments in abstinent alcohol patients

We investigated cognitive functioning following chronic alcohol consumption, in memory assessed by MFE scale and in the frontal lobe assessed by FAB scale, in plasma concentrations of total LPA and LPA species in the AUD group.

The AUD group showed a MFE score mean of 29.25 ± 16.47, indicating no deficits related in memory impairment. Moreover, a FAB score mean in AUD group was 15.25 ± 2.23, indicating a mild deficit in frontal lobe assessment (dysexecutive syndrome). Correlation analyses using Spearman correlation (rho) were performed in plasma concentrations of total LPA and LPA species with MFE and FAB scores, respectively (see Supplementary Table S6). In addition, a negative correlation was found between the MFE scores and FAB scores FAB scores (r = − 0.493, *p* < 0.001).

**Figure 2:**
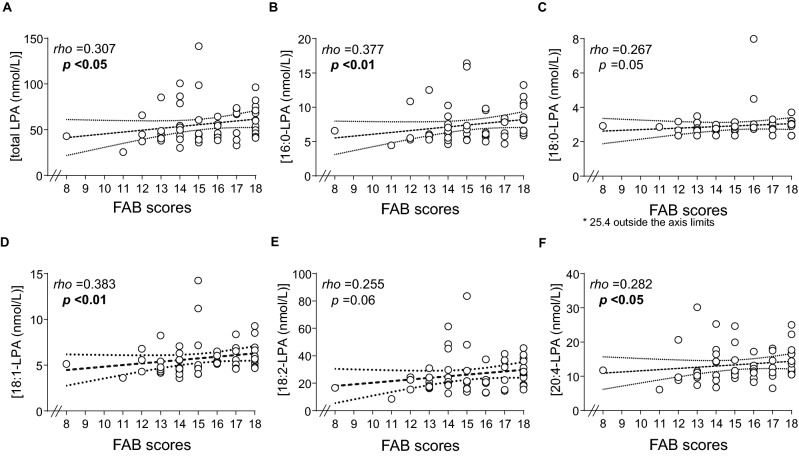
Correlations between LPA species and FAB scores in AUD group controlled by age and sex. (**A**) Total LPA (nmol/L) with FAB scores; (**B**) 16:0-LPA (nmol/L) with FAB scores; (**C**) 18:0-LPA (nmol/L) with FAB scores; (**D**) 18:1-LPA (nmol/L) with FAB scores; (**E**) 18:2-LPA (nmol/L) with FAB scores; (**F**) 20:4-LPA (nmol/L) with FAB scores. Dots are individual values. (rho) Spearman’s correlation coefficient; (p) p value for statistical significance.

As shown in Fig. 2, there were significant and positive correlations between the executive tasks evaluated with FAB scores and the plasma concentrations of total LPA, and the plasma concentrations of 16:0-LPA, 18:1-LPA and 20:4-LPA species. It is important to note that all the correlation analyses were double checked using a bootstrapped approach technique, and the Spearman correlation between total FAB and 18:0-LPA was not robust enough to be taken as a significant correlation result. Despite this fact, there were positive correlations between the executive tasks and other LPA species determined except for 18:0-LPA and 18:2-LPA. By contrast, we found no associations between memory impairments assessed with MFE and plasma concentrations of LPA species in the alcohol group. These data suggest a significant association between executive functions and circulating LPA species in the AUD group.

### Correlation of plasma concentrations of LPA species with growth factors

Moreover, correlation analyses using Pearson correlation (r) were performed with the logarithms of plasma concentrations of LPA (total LPA and LPA species) and growth factors (BDNF, IGF-1 and IGF-2, and NT-3) in the AUD group (Supplementary Table S7).

**Figure 3:**
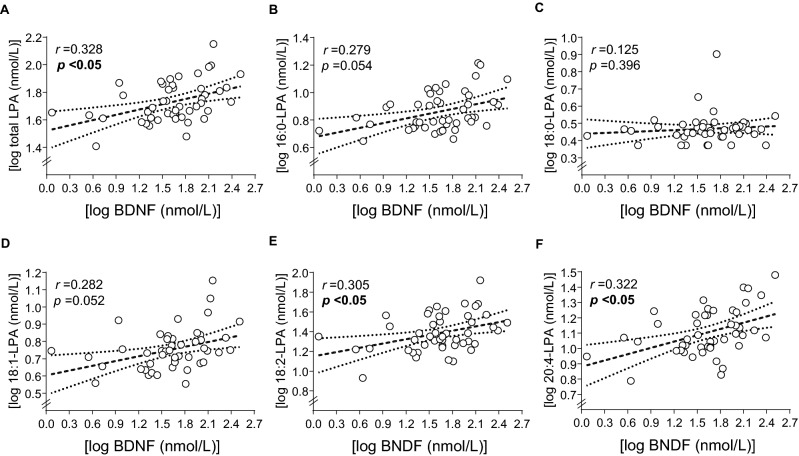
Correlations between LPA species and plasma concentrations of BDNF in AUD patients. (**A**) log BDNF (nmol/L) with log total LPA (nmol/L); (**B**) log BDNF (nmol/L) with log 16:0-LPA (nmol/L); (**C**) log BDNF (nmol/L) with log 18:0-LPA (nmol/L); (**D**) log 18:1-LPA (nmol/L) with log BDNF (nmol/L); (**E**) log BDNF (nmol/L) with log 18:2-LPA (nmol/L); (**F**) log BDNF (nmol/L) with log 20:4-LPA (nmol/L). Dots are individual values. (r) Pearson’s correlation coefficient; (p) p value for statistical significance.

As shown in Fig. 3, the statistical analyses found positive and significant correlations between plasma concentrations of BDNF and total LPA, 18:2-LPA and 20:4-LPA. However, there were no significant correlations between BDNF and 16:0-LPA, 18:0-LPA or 18:1-LPA.

**Figure 4:**
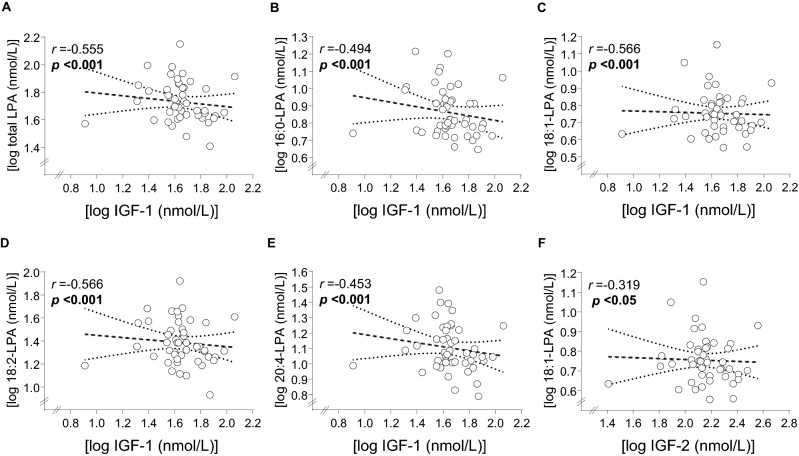
Significant correlations between LPA species and plasma concentrations of IGF-1 in AUD patients. (**A**) log IGF-1 (nmol/L) log with total LPA (nmol/L); (**B**) log IGF-1 (nmol/L) with log 16:0-LPA (nmol/L); (**C**) log IGF-1 (nmol/L) with log 18:1-LPA (nmol/L); (**D**) log IGF-1 (nmol/L) with log 18:2-LPA (nmol/L); (**E**) log IGF-1 (nmol/L) with log 20:4-LPA (nmol/L); (**F**) log IGF-2 (nmol/L) with log 18:1-LPA (nmol/L). Dots are individual values. (r) Pearson’s correlation coefficient; (p) p value for statistical significance.

Unlike BDNF, there were negative significant correlations between plasma concentrations of IGF-1 and IGF-2 plasma concentrations with some LPA species (Fig. 4). Specifically, IGF-1 correlated negatively with total LPA and all LPA species, except for 18:0-LPA; while IGF-2 only correlated negatively with 18:2-LPA.

## Discussion

Preclinical studies in animal models of alcohol dependence corroborate the participation of LPA and its LPA1 receptor in the spontaneous alcohol preference and alcohol drinking of mice, as well as in alcohol-associated changes in emotional memory and social/maternal behavior^34,42^. Another preclinical study using ethanol fed mice has described specific alterations on some fatty acids-related lipids^43^. These results are in agreement with the present study in AUD patients. Thus, our results showed that abstinent AUD patients displayed lower circulating levels of total LPA, 18:0-LPA and 18:1-LPA when compared with a control group. In addition to the present results, other studies have reported a deregulation of circulating fatty acids in AUD patients^41,44^, suggesting that fatty acid metabolism including other fatty-acid derived signals might be affected after alcohol exposure. Thus, it has been described higher concentrations of oleic acid in a group of patients with alcohol dependence that in the control group^44^. Following this rationale, we have recently described that AUD patients have elevated plasma concentrations of SEA and OEA, the stearic acid and oleic acid-derived acylethanolamides respectively involved in energy homeostasis and alcohol consumption^41,45^. Interestingly, we observed that total LPA and some LPA species, including 16:0-LPA, 18:2-LPA and 20:4-LPA, were affected by sex. Our results are in accordance with previous studies in healthy subjects that have reported sex differences with increased LPA concentrations in women^30,35^. However, we found differences in LPA concentrations between men and women in the control and AUD groups, suggesting that the presence of a sexual dimorphism in the circulating species of this lipid is not a specific factor associated to the impact of alcohol consumption.

Because our AUD sample displayed a high prevalence of SUDs (41.8%) and mental disorders (63.6%), we also evaluated the existence of a possible association between circulating LPA levels and the presence of SUDs and/or comorbid mental disorders. However, we did not find any significant association, maybe due to the small sample size. The elevated lifetime in psychiatric comorbid disorders in our sample is consistent with other reports in AUD populations^46,47^. Although we did not find significant differences in LPA plasma profiles between AUD patients with and without comorbid disorders, there is a growing body of evidence about the implication of fatty acids in certain mental disorders. Thus, serum levels of 16:0-LPA are upregulated in patients with major depressive disorders compared with a control group^48^. Other study show reduced plasma levels of arachidonic acid in bipolar depression patients^49^ and fatty acid deficiency in postmortem brain tissues samples^50^. In addition, the role of LPA species in the etiology of several neuropsychiatric disorders through the LPA1 receptor has also been examined both in human and preclinical models. The LPA deregulation has been studied in complex disorder such as schizophrenia in preclinical models^31,51^ and schizophrenia patients^52^. Finally, ATX as the primary biological source of LPA, represents a high-value psychiatric condition target^53^. ATX has been described as a possible biomarker of patients with major depression disorder diagnosis, since the serum levels of this enzyme are reduced in depressive patients compared with healthy controls^54^.

However, we found a clear association between LPA plasma concentrations and mild cognitive impairment. In our study, the AUD group displayed a mild deficit in tasks related to executive functions according with alcohol-related cognitive impairments. According to our results, executive functions are particularly affected in AUD population, although there are other neuropsychological processes including memory, emotional and psychosocial skills, visuospatial cognition and psychomotor impaired functions altered in alcohol dependence patients^55,56^. Other studies have reported increased difficulties in motivational processes in addiction treatment patients causing an underestimated impact on the efficacy and management on these clinical treatments^14,57^. Although there were no significant correlations between LPA plasma concentrations and memory impairments in our results, the negative correlation found between lobe function and memory scores, is consistent with early reports suggesting that general memory dysfunctions are related with other types of memory and to executive performance^58^. For that reason, it is of great interest the correlation found between 16:0-LPA, 18:0-LPA, 18:1-LPA and 20:4-LPA and the scores obtained in executive function assessment test. These results suggest that some LPA species might be good reliable markers for the detection of executive dysfunction associated with AUD. These findings support previous studies that have described a relation between dysfunctional levels of LPA signaling and neuropsychological impairments. For example, it has been reported that the plasma levels of LPA negatively correlated with mild cognitive impairments assessed with MoCA test in diabetic patients^59^. Regarding preclinical models, the lack of LPA1 receptor has been associated to cognitive alterations, using spatial memory tasks^60,61^. Moreover, a molecular study focused on lysophosphatidic acid acyltransferases (LPAATs) group of enzymes involved in the production of phosphatidic acid from LPA, shows that the inhibition of lipid metabolism is associated with physiological consequences such as cognitive dysfunction^62^.

In the present study, we also evaluated the correlation between the LPA species and growth factors. We found a positive correlation between total plasma concentrations of LPA (or that of polyunsaturated LPA) with circulating levels of the trophic factor BNDF, suggesting that both polyunsaturated LPA species and BDNF might contribute to normal cognitive processing. There is a clear association between the decrease in the circulating levels of these mediators, the impairment of cortical/hippocampal LPA and BDNF signaling, and alcohol associated cognitive impairment^18,19,29,42^. However, the mechanisms of this association are unknown. A potential interesting mechanism might be associated with neuroinflammatory processes. In this regard, previous studies with polyunsaturated fatty acids (i.e. arachidonic acid and their metabolites) have demonstrated a tight association between their ability to modulate both inflammation and BDNF production^63^.

Finally, we found that plasma levels of IGF-1 correlated negatively with total LPA, 16:0-LPA, 18:1-LPA, 18:2-LPA and 20:4-LPA and IGF-2 correlated negatively with 18:1-LPA. Previous studies have reported that insulin-like growth factors (i.e., IGF-1 and IGF-2) are associated with the maintenance of the cognitive functioning specially in attention and executive functions^64,65^. However since these growth factors are also decreased by alcohol, but they do not correlate with cognitive impairment or with comorbid mental disorders^17^, it is difficult to determine the nature of this association. One possible explanation might be derived of the shift on liver metabolism imposed by alcohol. Alcohol-associated cognitive impairment in the IGF-1 signaling could be a potential mechanism in the neuroinflammatory processes.

### Limitations of the present study

These finding described an important effect of alcohol consumption on LPA plasma concentrations, as well as an important association with executive functions and cognitive impairment. Moreover, we are aware about the existence of limitations in the present observational study. First, the recruitment of the sample was conducted from outpatient programs and there are uncontrolled social and environmental variables (e.g., diet, medication control) that could affect the validity of the results. Second, larger samples of male and female AUD patients and additional experimental groups should be included (e.g., patients with mental disorders but without substance use disorders for analyzing in depth the contribution of LPA to alcohol-induced brain damage). Third, longitudinal studies are also needed to monitor changes in these metabolites during abstinence at different times in the same patients. Finally, because a high percentage of AUD patients were under different pharmacological treatment, we cannot exclude the influence of specific medications on the circulating concentrations of the different LPA species.

## Conclusions

In agreement with previous preclinical studies supporting a role of the fatty acid related lipids and the lysophosphatidic acid receptor 1 (LPA1) in alcohol consumption^34,42,43^, the present results further suggest that LPA species are lipid mediators associated with AUD. The main findings of this study indicate that (a) The total circulating concentration of LPA, 16:0-LPA, 18:0-LPA and 18:1-LPA were decreased in the AUD group when compared to the control group; (b) The total circulating concentration of LPA, 16:0-LPA, 18:2-LPA and 20:4-LPA were decreased in men compared to women (c) The plasma concentrations of the LPA species were not significantly affected by the presence of lifetime comorbid mood and anxiety disorders; (d) In the AUD group, 22% of the patients had cognitive deficits related to executive functions, while 32.6% displayed deficits related to memory impairments; (e) The executive tasks scores of the AUD group correlated with plasma concentrations of total LPA, 16:0-LPA, 18:1-LPA and 20:4-LPA; (f) There is a clear positive correlation between plasma concentrations of BDNF and total LPA, 18:2-LPA and 20:4-LPA; (g) There is a strong inverse correlation between IGF-1 and total LPA, 16:0-LPA, 18:1-LPA, 18:2-LPA and 20:4-LPA. Overall, these data suggest that LPA species are affected by chronic alcohol consumption, and they are associated with cognitive impairments similar to trophic factors such as BDNF^18^.

## Supplementary information

Supplementary Information.
