# Supplementary material for: Potential association of plasma lysophosphatidic acid (LPA) species with cognitive impairment in abstinent alcohol use disorders outpatients
Source: Sci Rep. 2020 Oct 13;10:17163. doi: 10.1038/s41598-020-74155-0 (PMC7555527; doi:10.1038/s41598-020-74155-0)
Supplement: Supplementary file 1 — Supplementary Information. [file 41598_2020_74155_MOESM1_ESM.docx]

**SUPPLEMENTARY MATERIAL**

**Potential association of plasma lysophosphatidic acid (LPA) species with cognitive impairment in abstinent alcohol use disorders outpatients**

Nuria García-Marchena^1,2#^*; Nieves Pizarro^3#^; Francisco J. Pavón^1^; Miriam Martínez-Huélamo^3,4^; María Flores-López^1^; Nerea Requena-Ocaña^1^; Pedro Araos^5^; Daniel Silva-Peña^1^; Juan Suárez^1^; Luis J. Santín^5^; Rafael de la Torre^3^*; Fernando Rodríguez de Fonseca^1^*; Antonia Serrano^1^*.

^1^Unidad Gestión Clínica de Salud Mental, Instituto de Investigación Biomédica de Málaga (IBIMA), Hospital Regional Universitario de Málaga/Universidad de Málaga, Málaga, Spain

^2^ Institut Germans Trias i Pujol (IGTP). Campus Can Ruti, Badalona, Spain.

^3^Programa de Investigación en Neurociencias, Institut Hospital del Mar d’Investigacions Mediques(IMIM), Barcelona, Spain

^4^Departamento de Nutrición, Ciencias de los Alimentos y Gastronomía. Facultad de Farmacia y Ciencias de los Alimentos, Universidad de Barcelona, Barcelona, Spain

^5^Departamento de Psicobiología y Metodología de las Ciencias del Comportamiento, Instituto de Investigación Biomédica de Málaga (IBIMA), Facultad de Psicología, Universidad de Málaga (UMA), Málaga, Spain

**Supplementary Table** S1: Analytes and selected reaction monitoring (SRM) conditions for the detection of LPA species in human plasma

| **Analyte** | **Precursor (m/*z*)** | **Product (m/*z*)** | **Collision energy (eV)** |
| --- | --- | --- | --- |
| **16:0 LPA** | 409 | 79 | 40 |
|  |  | 153 | 20 |
| **17:0 LPA*** | 423 | 79 | 50 |
|  |  | 153 | 20 |
| **18:0 LPA** | 437 | 79 | 40 |
|  |  | 153 | 20 |
| **18:1 LPA** | 435 | 79 | 50 |
|  |  | 153 | 30 |
| **18:2 LPA** | 433 | 79 | 50 |
|  |  | 153 | 20 |
| **20:4 LPA** | 457 | 79 | 40 |
|  |  | 153 | 20 |

**Supplementary Table S2:** Plasma concentrations of LPA species grouped according to history of AUD.

| **VARIABLE** | **Control group**  **(N=34)** | **AUD group**  **(N=55)** | **Statistics^(1)^** | | |
| --- | --- | --- | --- | --- | --- |
|  |  |  | **F-value** | **df** | ***p*-value** |
|  | **Mean [95%CI]** | **Mean [95%CI]** |  |  |  |
| **Total LPA**  **(nmol/L)** | 74.922  [65.190-84.512] | 60.847  [52.190-69.503] | 4.629 | 1,82 | **0.034** |
| **16:0 LPA**  **(nmol/L)** | 10.096  [8.861-11.332] | 8.094  [6.979-9.210] | 5.640 | 1,82 | **0.020** |
| **18:0 LPA**  **(nmol/L)** | 3.372  [3.105-3.639] | 2.958  [2.717-3.199] | 5.166 | 1,82 | **0.026** |
| **18:1 LPA**  **(nmol/L)** | 7.489  [6.670-8.308] | 5.998  [5.258-6.738] | 7.114 | 1,82 | **0.009** |
| **18:2 LPA**  **(nmol/L)** | 37.011  [31.278-42.744] | 29.430  [24.255-34.606] | 3.756 | 1,82 | 0.056 |
| **20:4 LPA**  **(nmol/L)** | 16.954  [14.558-19.350] | 14.366  [12.204-16.529] | 2.507 | 1,82 | 0.117 |

Abbreviations: LPA= lysophosphatidic acid; df = degree of freedom. **^(1)^** Two-way ANCOVA was performed using lifetime AUD and sex as factors and controlling for age and BMI as covariates.

**Supplementary Table S3:** Plasma concentrations of LPA species grouped according to sex.

| **VARIABLE** | **Women**  **(N=34)** | **Men**  **(N=55)** | **Statistics^(1)^** | | |
| --- | --- | --- | --- | --- | --- |
|  |  |  | **F-value** | **df** | ***p*-value** |
|  | **Mean [95%CI]** | **Mean [95%CI]** |  |  |  |
| **Total LPA**  **(nmol/L)** | 77.360  [66.023-88.698] | 58.409  [52.205-64.613] | 8.377 | 1,82 | **0.005** |
| **16:0 LPA**  **(nmol/L)** | 10.465  [9.004-11.926] | 7.726  [6.926-8.525] | 10.539 | 1,82 | **0.002** |
| **18:0 LPA**  **(nmol/L)** | 3.213  [2.897-3.528] | 3.117  [2.945-3.290] | 0.272 | 1,82 | 0.405 |
| **18:1 LPA**  **(nmol/L)** | 7.266  [6.297-8.235] | 6.221  [5.691-6.751] | 3.492 | 1,82 | 0.065 |
| **18:2 LPA**  **(nmol/L)** | 39.012  [32.234-45.790] | 27.429  [23.720-31.138] | 8.755 | 1,82 | **0.004** |
| **20:4 LPA**  **(nmol/L)** | 17.405  [14.572-20.237] | 13.916  [12.366-15.466] | 4.548 | 1,82 | **0.036** |

Abbreviations: LPA= lysophosphatidic acid; df=degree of freedom.**^(1)^** Two-way ANCOVA was performed using lifetime AUD and sex as factors and controlling for age and BMI as covariates.

**Supplementary Table S4:** Plasma concentrations of LPA species grouped according to comorbid mood disorders.

| **AUD group (N=55)** | | | | | |
| --- | --- | --- | --- | --- | --- |
| **VARIABLE** | **Comorbid mood disorders**  **(N=24)** | **No mood disorders**  **(N=31)** | **Statistics^(1)^** | | |
|  |  |  | **F-value** | **df** | ***p*-value** |
|  | **Mean [95%CI]** | **Mean [95%CI]** |  |  |  |
| **Total LPA** | 56.673  [46.759-66.586] | 64.991  [50.808-79.175] | 0.816 | 1,49 | 0.358 |
| **16:0 LPA**  **(nmol/L)** | 7.642  [6.402-8.882] | 8.364  [6.590-10.138] | 0.415 | 1,49 | 0.523 |
| **18:0 LPA**  **(nmol/L)** | 2.819  [2.435-3.203] | 3.143  [2.594-3.692] | 0.871 | 1,49 | 0.355 |
| **18:1 LPA**  **(nmol/L)** | 5.666  [4.782-5.309] | 6.574  [5.309-7.840] | 1.288 | 1,49 | 0.262 |
| **18:2 LPA**  **(nmol/L)** | 32.791  [23.718-41.864] | 26.736  [20.394-33.078] | 1.115 | 1,49 | 0.296 |
| **20:4 LPA**  **(nmol/L)** | 13.809  [11.509-16.109] | 14.118  [11.509-16.109] | 0.022 | 1,49 | 0.882 |

Abbreviations: LPA= lysophosphatidic acid; df = degree of freedom. **^(1)^**Two-way ANCOVA was performed using lifetime mood disorders and sex as factors and controlling for age and BMI as covariates.

**Supplementary Table S5:** Plasma concentrations of LPA species grouped according to comorbid anxiety disorders.

| **AUD group (N=55)** | | | | | |
| --- | --- | --- | --- | --- | --- |
| **VARIABLE** | **Comorbid anxiety disorders**  **(N=19)** | **No anxiety disorders**  **(N=36)** | **Statistics^(1)^** | | |
|  |  |  | **F-value** | **df** | ***p*-value** |
|  | **Mean [95%CI]** | **Mean [95%CI]** |  |  |  |
| **Total LPA** | 61.127  [50.351-71.902] | 58.085  [45.862-70.308] | 0.136 | 1,49 | 0.714 |
| **16:0 LPA**  **(nmol/L)** | 8.045  [6.698-9.391] | 7.930  [6.402-9.458] | 0.012 | 1,49 | 0.912 |
| **18:0 LPA**  **(nmol/L)** | 2.823  [2.412-3.235] | 3.035  [2.569-3.502] | 0.454 | 1,49 | 0.504 |
| **18:1 LPA**  **(nmol/L)** | 6.136  [5.174-7.097] | 5.916  [4.825-7.007] | 0.089 | 1,49 | 0.766 |
| **18:2 LPA**  **(nmol/L)** | 30.063  [23.202-36.924] | 27.172  [19.389-34.955] | 0.303 | 1,49 | 0.584 |
| **20:4 LPA**  **(nmol/L)** | 14.060  [11.559-16.561] | 14.031  [11.194-16.869] | 0.000 | 1,49 | 0.988 |

Abbreviations: LPA= lysophosphatidic acid; df=degree of freedom. **^(1)^**Two-way ANCOVA was performed using lifetime anxiety disorders and sex as factors and controlling for age and BMI as covariates.

| **VARIABLE** | **Total LPA**  **(nmol/L)** | **16:0 LPA**  **(nmol/L)** | **18:0 LPA**  **(nmol/L)** | **18:1 LPA (nmol/L)** | **18:2 LPA**  **(nmol/L)** | **20:4 LPA**  **(nmol/L)** |
| --- | --- | --- | --- | --- | --- | --- |
|  | **rho** | **rho** | **rho** | **rho** | **rho** | **rho** |
|  | ***p*-value** | ***p*-value** | ***p*-value** | ***p*-value** | ***p*-value** | ***p*-value** |
| **Total MFE**  **scores** | -0.030 | -0.075 | -0.195 | -0.147 | -0.038 | -0.005 |
|  | 0.827 | 0.587 | 0.154 | 0.285 | 0.783 | 0.969 |
| **Total FAB**  **scores** | 0.307 | 0.377 | 0.277 | 0.383 | 0.255 | 0.282 |
|  | **0.023** | **0.005** | **0.049** | **0.004** | 0.060 | **0.037** |

**Supplementary Table S6:** Correlation between LPA species with cognitive impairment scores in AUD group.

Abbreviations: MFE= memory failure everyday; FAB= frontal assessment battery; rho= Spearman’s correlation coefficient.

| **VARIABLE** | **Log total LPA**  **(nmol/L)** | **Log 16:0 LPA**  **(nmol/L)** | **Log 18:0 LPA**  **(nmol/L)** | **Log 18:1 LPA**  **(nmol/L)** | **Log 18:2 LPA**  **(nmol/L)** | **Log 20:4 LPA**  **(nmol/L)** |
| --- | --- | --- | --- | --- | --- | --- |
|  | **r** | **r** | **r** | **r** | **r** | **r** |
|  | ***p*-value** | ***p*-value** | ***p*-value** | ***p*-value** | ***p*-value** | ***p*-value** |
| **Log BDNF**  **(nmol/L)** | 0.328 | 0.279 | 0.125 | 0.282 | 0.305 | 0.322 |
|  | **0.023** | 0.054 | 0.396 | 0.052 | **0.035** | **0.026** |
| **Log IGF-1**  **(nmol/L)** | -0.555 | -0.494 | 0.105 | -0.566 | -0.566 | -0.453 |
|  | **<0.001** | **<0.001** | 0.477 | **<0.001** | **<0.001** | **0.001** |
| **Log IGF-2**  **(nmol/L)** | -0.216 | -0.115 | 0.129 | -0.319 | -0.254 | -0.111 |
|  | 0.141 | 0.437 | 0.382 | **0.027** | 0.082 | 0.452 |
| **Log NT-3**  **(nmol/L)** | 0.240 | 0.244 | 0.193 | 0.213 | -0.244 | 0.157 |
|  | 0.101 | 0.095 | 0.190 | 0.145 | 0.095 | 0.286 |

**Supplementary Table S7:** Correlation of plasma concentrations between LPA species with growth factors in AUD group.

Abbreviations: r= Pearson’s correlation coefficient.
